# Accuracy and reliability of measurements obtained with a noncontact tono-pachymeter for clinical use in mass screening

**DOI:** 10.1038/s41598-021-88364-8

**Published:** 2021-04-26

**Authors:** Jinho Lee, Hyuk Jin Choi

**Affiliations:** 1 Department of Ophthalmology, Seoul National University College of Medicine, 103 Daehak-ro, Jongno-gu, Seoul, 03080 Korea; 2 Department of Ophthalmology, Hallym University Chuncheon Sacred Heart Hospital, Chuncheon, Korea; 3 Seoul National University Hospital Healthcare System Gangnam Center, Seoul, Korea

**Keywords:** Glaucoma, Population screening

## Abstract

We evaluated the reliability and accuracy of the noncontact CT-1P tonopachymeter (Topcon, Japan) in terms of intraocular pressure (IOP) and central corneal thickness (CCT). One hundred sixty-three healthy participants and 33 patients with open angle glaucoma were enrolled. IOPs were measured by CT-1P (T-IOP) and Goldmann applanation tonometer (G-IOP), and CCTs were measured by the CT-1P (T-CCT) and an ultrasound pachymeter (US-CCT). Both CCT instrument-adjusted (T-IOP-C) and unadjusted T-IOPs (T-IOP-NC) were included. Pearson correlation coefficients and biases assessed with Bland–Altman analysis with 95% confidence interval (CI) were calculated for reliability evaluation. Intrasession repeatability was excellent for both T-IOP (intraclass correlation coefficient [ICC] 0.91) and T-CCT (ICC 0.98). Intersession reproducibility was also excellent for T-CCT (ICC 0.94). T-IOP-NC and T-IOP-C both showed significant correlations with G-IOP (r = 0.801, *P * <  0.001 and r = 0.658, *P * < 0.001, respectively). T-CCT was also strongly correlated with US-CCT (r = 0.958; *P * < 0.001). T-IOP-NC and T-IOP-C both showed a positive bias (1.37 mmHg, 95% CI [1.14, 1.61] and 2.77 mmHg, 95% CI [2.49, 3.05], respectively). T-CCT showed a negative bias of − 17.3 µm (95% CI [−18.8, − 15.8]). With cautious interpretation, the CT-1P may offer good feasibility for IOP and CCT measurement in screening centers.

## Introduction

In glaucoma, accurate measurements of intraocular pressure (IOP) are crucial for monitoring and managing the disease course. Goldmann applanation tonometry (GAT) has been considered the gold standard method of IOP measurement^1–3^. However, it has some unignorable drawbacks compared with noncontact tonometers (NCTs). First, topical anesthetics and fluorescein dye are needed before measurement, which are prone to allergic reaction^4^ or other patient discomforts. Second, the necessity of slit lamp biomicroscopy makes IOP measurements more complicated than NCTs. In contrast, NCT is a relatively handy and simple method for IOP measurement that can be performed by ancillary staff without the use of corneal anaesthesia^5^.

Central corneal thickness (CCT) is another an important barometer for glaucoma monitoring. It is well known that CCT significantly affects IOP measurement^6,7^. Moreover, CCT can be a predictive factor for glaucoma progression in ocular hypertension^8,9^. Among various instruments for CCT measurement, ultrasound (US) pachymetry has been widely considered the gold standard method by virtue of its easy and fast acquisition and good repeatability^10,11^. However, it also has some disadvantages: (i) direct placement of the probe on the cornea, which is susceptible to infection and corneal epithelial damage, (ii) the necessity for topical anesthesia, and (iii) dependence on examiner experience for reliable measurements^12^.

Tono-pachymeters, which have recently become commercially available, simultaneously measure CCT using the principle of the Scheimpflug camera system and IOP using a conventional noncontact tonometry method. Tono-pachymetry is patient-friendly and time-saving and can be performed by well-trained ancillary staff. Thus, it can be suitable for the mass screening of glaucoma. However, the reliability and agreement with conventional gold standard methods need to be verified.

The Topcon CT-1P (Topcon Inc., Tokyo, Japan) is a fully automated, noncontact tono-pachymeter that provides noncontact measurements of IOP. CCT can also be measured using the pachymetry feature, and the instrument automatically provides a CCT-adjusted IOP. There are a few previous reports that have documented the reliability of the CT-1P and its comparison with other IOP or CCT measurement methods. According to Bang et al., a significant positive correlation was shown between the IOP values obtained with GAT and the CT-1P, but the IOP measured with CT-1P tended to be higher than that measured with GAT (mean bias = 0.48 mmHg)^13^. In terms of CCT, the CT-1P tono-pachymeter tended to underestimate CCT measurements with respect to those of the Scheimpflug system, anterior segment optical coherence tomography (AS-OCT) device, and US pachymetry^14^. To the best of our knowledge, however, there are no reports investigating the repeatability and reliability of this tono-pachymeter.

The purpose of this study was to evaluate the repeatability, reproducibility, and accuracy of the CT-1P with regard to IOP and CCT measurements and to compare these measurements with those obtained from GAT and US pachymetry.

## Results

### Subject demographics

A total of 196 eyes from 196 subjects were included in this study. Among them, 163 subjects were healthy controls enrolled from a glaucoma screening program, and 33 glaucoma patients were recruited from the Glaucoma Outpatient Clinic at the Seoul National University Hospital (SNUH) Healthcare System Gangnam Center (HSGC). The primary open angle glaucoma (POAG) group (mean age: 55.0 ± 8.0 years) was significantly older than normal group (mean age: 51.7 ± 11.2; P = 0.046). Eighty-six women (43.8%) and 110 men were recruited; the sex difference was not significantly different between the two groups (P = 1.000). There were no significant differences in the IOP measured by the CT-1P without (T-IOP-NC) or with correction for CCT (T-IOP-C), the IOP measured by GAT (G-IOP), the CCT measured by the CT- 1P (T-CCT), or the CCT measured by US pachymetry (US-CCT) between the two groups. A detailed description of the clinical characteristics of the study population is provided in Table 1.

**Table 1:** Basic clinical characteristics of the study population.

	Normal	Glaucoma	P
No	163 patients (163 eyes)	33 patients (33 eyes)	N/A
Age (years)	51.7 ± 11.2	55.0 ± 8.0	0.046
Female (%)	72 (44.2%)	14 (42.4%)	1.000
T-IOP-NC (mmHg)	15.4 ± 2.7	15.0 ± 2.7	0.449
T-IOP-C (mmHg)	16.6 ± 2.2	16.9 ± 2.2	0.486
G-IOP (mmHg)	13.8 ± 2.6	14.4 ± 2.3	0.244
T-CCT (µm)	518.9 ± 36.7	505.6 ± 30.4	0.052
US-CCT (µm)	536.1 ± 37.4	523.7 ± 28.3	0.073

T-IOP-NC: intraocular pressure (IOP) measured by Topcon CT-1P without correction of central corneal thickness (CCT); T-IOP-C: IOP measured by Topcon CT-1P with CCT correction; G-IOP: IOP measured by Goldmann applanation tonometer; T-CCT: CCT measured by Topcon CT-1P; US-CCT: CCT measured by ultrasound pachymetry.

### Repeatability and reproducibility

The mean and standard deviation (SD) of the first and second T-IOP-NC values acquired were 15.1 ± 2.8 mmHg and 15.2 ± 2.8 mmHg, respectively. The mean and SD of the first and second CCTs acquired were 517.1 ± 35.9 μm and 516.7 ± 36.5 μm, respectively. The ICC values for T-IOP-NC and T-CCT were 0.91 (95% CI [0.89, 0.92]; P < 0.001) and 0.98 (95% CI [0.98, 0.98]; P < 0.001), respectively. Both T-IOP-NC and CCT showed excellent intrasession repeatability. Considering the physiological IOP fluctuations, intersession reproducibility was evaluated only for T-CCT. For the 140 patients who had undergone T-CCT measurement 3 times, intersession reproducibility was excellent (ICC 0.94, 95% CI [0.93, 0.95]; P < 0.001). The coefficient of variation (CoV) values for CCT was 1.47%, which was excellent. The Bland–Altman plots and scatterplots of T-IOP-NC and T-CCT for intrasession repeatability are provided in Supplemental Figure S1 and S2.

### Comparison of the CCT and IOP values measured by CT-1P with the gold standards

**Figure 1:**
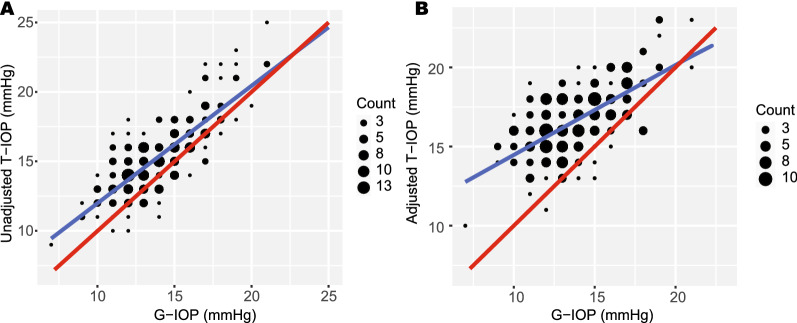
Scatterplot showing the correlation between intraocular pressure (IOP) measurements taken with a Topcon CT-1P noncontact tonopachymeter (T-IOP) and a Goldmann applanation tonometer (G-IOP). The least square regression lines are shown as blue, whilst the identity lines (where G-IOP equals to T-IOP) are plotted as red lines. Significant positive correlations were observed **(A)** before adjusting T-IOP (Pearson correlation coefficient r = 0.801; P < 0.001) and **(B)** after adjusting T-IOP for central corneal thickness (r = 0.658; P < 0.001). Note that majority of the dots are located above the identity line, meaning that T-IOPs were generally higher than G-IOPs.

**Figure 2:**
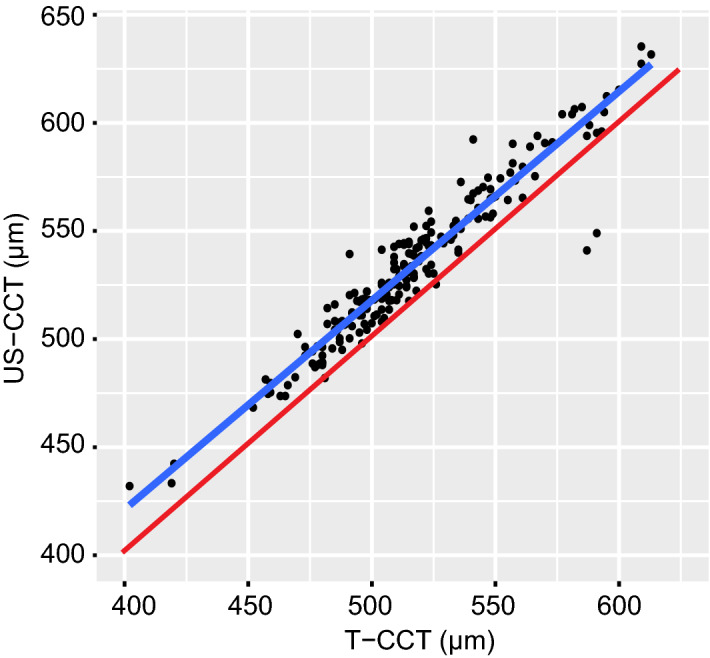
Scatterplot revealing the correlation between central corneal thickness (CCT) measurements taken with the Topcon CT-1P noncontact tonopachymeter (T-CCT) and ultrasound pachymetry (US-CCT). The least square regression line is shown as blue, and the identity line (where US-CCT equals to T-CCT) is plotted as a red line. A strong positive correlation was observed, with a Pearson correlation coefficient of 0.958 (P < 0.001).

A strong correlation was shown between T-IOP-NC and G-IOP (r = 0.801, P < 0.001; Fig. 1A). A significantly positive but moderate correlation was found between T-IOP-C and G-IOP (r = 0.658, P < 0.001; Fig. 1B). The correlation of the CCT value between the CT-1P and ultrasound pachymetry was found to be very strong (r = 0.958, P < 0.001; Fig. 2).

The mean values of T-IOP-NC and G-IOP were 15.3 ± 2.7 mmHg and 13.9 ± 2.6 mmHg, respectively. The IOP acquired by the CT-1P was significantly higher than G-IOP (P < 0.001). In most cases (136 eyes, 69.4%), the IOPs measured by the CT-1P were higher than those obtained by GAT. The mean values of T-CCT and US-CCT were 516.7 ± 36.0 mmHg and 533.9 ± 36.3 mmHg, respectively. The CCT acquired by the CT-1P was significantly lower than US-CCT (P < 0.001). T-CCT was lower than US-CCT for nearly all subjects (193 eyes, 98.5%).

**Figure 3:**
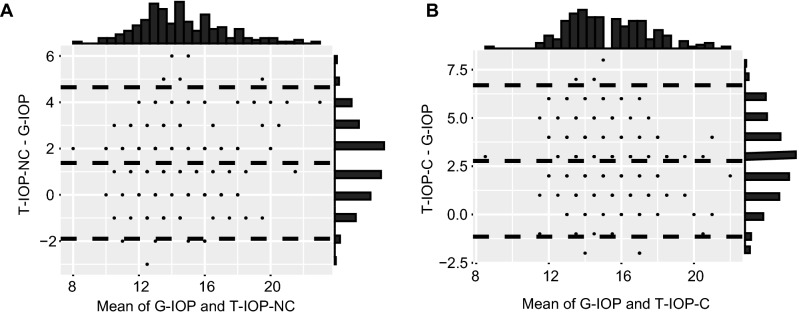
Bland–Altman plots between the IOP measurements of the Topcon noncontact tonopachymeter and Goldmann applanation tonometry (G-IOP). The dashed line in the center indicates the mean difference between both tonometers. The upper and lower dashed lines are the 95% limits of agreement (LoA). **(A)** Before adjusting IOP for CCT (T-IOP-NC), the mean bias was 1.37 mmHg (95% CI [1.14, 1.61]; 95% LoA [-1.89, 4.65 mmHg]). **(B)** After adjusting IOP for CCT (T-IOP-C), the mean bias was 2.77 mmHg (95% CI [2.49, 3.05]; 95% LoA [-1.14, 6.69 mmHg]). IOP = intraocular pressure; CCT = central corneal thickness; CI = confidence interval.

A Bland–Altman plot comparing the T-IOP-NC and G-IOP readings (Fig. 3A) showed reasonable agreement between the methods. The mean IOP difference was 1.37 mmHg (95% CI [1.14, 1.61]), and the 95% limit of agreement (LoA) was -1.89 to 4.65 mmHg. These differences did not vary proportionally to the mean of the two measurement values. In 76.5% of subjects, the IOP difference between the two tonometry readings was ≤ 2 mmHg, and in 88.8% of subjects, it was ≤ 3 mmHg. When comparing the T-IOP-C and G-IOP, the mean bias was found to be 2.77 mmHg (95% CI [2.49, 3.05]), and the 95% LoA was -1.14 to 6.69 mmHg (Fig. 3B). That is, the CCT-adjusted T-IOP tended to be more overestimated with respect to G-IOP than T-IOP-NC.

**Figure 4:**
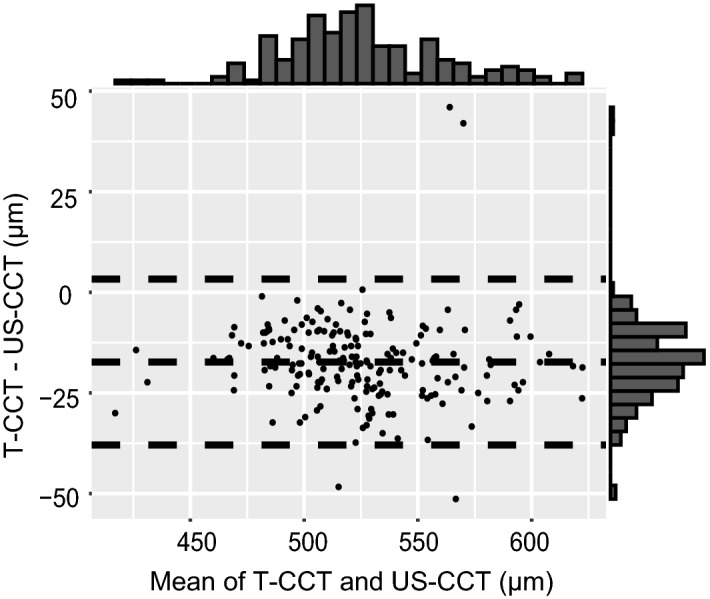
Bland–Altman plots between the CCT measurements of the Topcon noncontact tonopachymeter (T-CCT) and the US pachymeter (US-CCT). The dashed line in the center indicates the mean difference between the two devices. The upper and lower dashed lines are the 95% limits of agreement (LoA). The mean bias was -17.3 µm (95% CI [-18.8, -15.8]; 95% LoA [-37.9, 3.30 µm]). CCT = central corneal thickness; US = ultrasound; CI = confidence interval.

In the Bland–Altman plot between the T-CCT and US-CCT measurements, T-CCT was generally undervalued than US-CCT. The mean CCT difference was -17.3 µm (95% CI [− 18.8, − 15.8]), and the 95% LoA was − 37.9 to 3.30 µm (Fig. 4). In 86.7% of subjects, the relative error (CCT difference/US-CCT) was ≤ 5%.

## Discussion

In the present study, the repeatability, reproducibility and accuracy of the IOP and CCT values observed with the CT-1P were evaluated. The results revealed excellent intrasession repeatability (ICC of IOP: 0.91, ICC of CCT: 0.98) and intersession reproducibility (ICC of CCT: 0.94). The IOP and CCT values obtained with the CT-1P showed strong positive correlations with the values obtained with GAT and US pachymetry (r = 0.801 and 0.958, respectively). Additionally, T-IOP-NC showed good agreement with G-IOP. T-CCT was generally lower than US-CCT, but in most cases (86.7%), the difference was within the clinically acceptable range (≤ 5%).

NCTs such as the Topcon CT-1P have some advantages for mass screening compared to GAT, such as the unnecessariness of corneal anaesthesia^5^. However, to investigate the feasibility of the device for glaucoma screening, the measurements from the device need to show small test–retest variability. First, regarding IOP, the intraobserver repeatability of the CT-1P was comparable to that of other noncontact tonometers^15,16^ and was even better than that of the iCare rebound tonometer (ICC 0.73–0.82)^17,18^. The CCT measurement also showed excellent repeatability and reproducibility, which was similar to those of other instruments. The repeatability for CCT was reported to be 0.992 for optical low-coherence reflectometry (Lenstar LS 900; Haag Streit, Köniz, Switzerland)^19^. A previous study comparing partial coherence interferometry (PCI) and 3 US pachymeters demonstrated that the ICC for intraobserver variability was 0.999 for PCI versus 0.987–0.995 for US^20^.

In Pearson correlation analysis, a significant, strong correlation was observed between T-IOP-NC and G-IOP and between T-CCT and US-CCT. The correlation coefficient of the CT-1P for IOP was similar to those of previously introduced NCTs^13,21^. The correlation between T-CCT and US-CCT is also comparable to that of a previous study (r = 0.857)^22^. This suggests that the IOP and CCT observed by the Topcon CT-1P can provide significant predictions for G-IOP and US-CCT.

Many ocular or systemic factors, including tear film height, astigmatism, corneal thickness, corneal hysteresis, or arrhythmia, affect IOP measurement^23^. Considering such variabilities, IOP measurement error may be clinically acceptable up to 3 mmHg^24^. In the study population, 76.5% showed IOP differences between the two tonometry readings ≤ 2 mmHg, and 88.8% showed IOP differences ≤ 3 mmHg. Based on this result, we could assume that IOP measurements from the CT-1P showed good agreement with those obtained with GAT. However, IOP measurements with the CT-1P tended to be slightly higher than G-IOP (mean bias = 1.37 mmHg). This finding is consistent with a previous report^13^, in which the mean difference between T-IOP and G-IOP was 0.48 ± 2.12 mmHg. In healthy subjects, the mean IOP measured by the CT-1P (15.4 ± 2.7 mmHg) was similar to previously reported values (15–16 mmHg on average, SD 2.5–3.0 mmHg)^6,25^. T-IOP-C was higher than T-IOP-NC in most cases, which accompanied a larger mean bias to G-IOP than T-IOP-NC. This is attributable to the underestimation of CCT by the CT-1P. In addition, the correlation between T-IOP-NC and G-IOP was stronger than that of T-IOP-C and G-IOP. It is well known that IOP measurements are affected by CCT^6,7^. Adjusted IOP is calculated according to the formula: *Adjusted IOP* = *measured IOP* + *(Standard CCT − measured CCT* × *Coefficient of adjustment)*^26^. Therefore, the dependency on CCT has been eliminated in T-IOP-C, explaining the weaker correlation than that of T-IOP-NC.

The mean difference between T-CCT and US-CCT was relatively larger than that between T-IOP and G-IOP and had a distinct tendency toward underestimation. We found two previous works in line with our conclusion for CCT underestimation by the CT-1P compared to US pachymetry^14,22^. There are a few possible reasons for these differences. Since a probe has to reach the cornea perpendicularly, topical anesthetics are needed for US pachymetry. It can affect at most 10 μm of the CCT measurement^12,27^. Furthermore, the US-CCT value can be dependent on the speed in tissues of variable environments and on different levels of examiner experience^28^. In contrast, the noncontact tono-pachymeter avoids contact with the cornea and uses light reflection through the front and back of the cornea. Because the principles used to delimit the front and backside of the cornea of the CT-1P are different from those of the US pachymeter, this discrepancy may play a role in the difference in the measured CCT. Although the absolute error was within the acceptable range in the majority of cases, clinicians should be careful when interpreting T-CCT.

There are some limitations in this study. First, all the subjects were from a Korean population, and the number of glaucoma patients was relatively smaller than the number of normal subjects. We determined that the reliability of this tono-pachymeter did not significantly differ between the two groups; however, further research with more glaucoma patients is warranted. Second, the repeatability was evaluated with only a single observer in a short period of time. The measurements from different technicians may show larger variability. Last, we did not consider the CCT fluctuation when calculating the intersession reproducibility. CCT also has diurnal fluctuations like IOP, although the amount of variability is reported to be small^29,30^.

In conclusion, the Topcon CT-1P noncontact tono-pachymeter showed good repeatability and agreement with GAT and ultrasound pachymetry. With cautious interpretation, it can be a useful tool in health screening centers.

## Methods

### Subjects

The present study included subjects from the *Gangnam Eye Cohort Study*, an ongoing cohort study conducted by SNUH HSGC. Detailed information on this cohort has been published elsewhere^31^. The present study was approved by the Institutional Review Board (IRB) of SNUH (IRB No. 1906-141-1043) and followed the tenets of the Declaration of Helsinki. Written informed consent was obtained from all subjects.

The study population comprises healthy subjects who had participated in a glaucoma screening program at the SNUH HSGC and patients diagnosed with POAG at the Glaucoma Outpatient Clinic of SNUH HSGC. The inclusion criteria for healthy subjects were participation in a glaucoma-screening program at the SNUH HSGC during the period from January 2017 to December 2018 with age > 40 years at the time of the first exam.

Individuals identified for exclusion showed (1) a secondary cause of glaucomatous optic neuropathy, (2) ocular or systemic disease that may cause visual field (VF) loss or other optic disc abnormalities, and (3) a history of intraocular surgery other than uncomplicated cataract surgery. One eye was randomly chosen from each patient for statistical analysis.

### Glaucoma-screening program

The glaucoma-screening examination comprised IOP measurement by a noncontact tono-pachymeter (model CT-1P; Topcon Inc., Tokyo, Japan) along with GAT (model AT900; Haag-Streit, Köniz, Switzerland) and fundus photography by a nonmydriatic fundus camera (model TRC-NW8, Topcon Inc., Tokyo, Japan). The fundus photographs were evaluated by an experienced ophthalmologist (HJC) for suspicious findings such as glaucomatous optic nerve head (ONH) changes or retinal nerve fiber layer (RNFL) defects. Subjects with suspected glaucomatous optic neuropathy, suspected RNFL defects, or IOP > 21 mmHg were referred for definite glaucoma examination.

### IOP and CCT measurements

In a fixed sequence, all of the subjects were examined with a CT-1P noncontact tono-pachymeter (NCT) and ultrasound pachymeter (Pocket II; Quantel Medical, Clermont-Ferrand, France), followed by GAT to obtain IOP and central corneal thickness (CCT) measurements. Since cornea compression during GAT acquisition can induce an increase in aqueous outflow, which might affect subsequent IOP measurements, Goldman IOP was obtained after NCT acquisition^32,33^. NCT and CCT measurements were made by the same experienced ancillary staff. IOP measured with the Topcon CT-1P (T-IOP) was recorded with or without adjustment by the CCT instrument. CCT was recorded from the CT-1P and from the US pachymeter for comparison.

GAT measurements were taken with an AT900 according to the standard procedures. One drop of 0.5% proparacaine hydrochloride eye drops (Paracaine, Alcon Laboratories Inc., Fort Worth, TX, USA) was instilled before acquisition, and a fluorescein strip was applied to the inferior conjunctival fornix. To avoid errors introduced by the topical anesthesia, G-IOP was obtained five minutes after eyedrop instillation^34^. All GAT measurements were obtained by the same experienced ophthalmologist (HJC), and the mean of the three GAT measurements was used for analysis.

Each tonometer was calibrated according to the manufacturer’s guidelines prior to its use in this study. Between each instrumentation application, the subjects were allowed a five-minute rest period to recover from the aqueous outflow.

### Diagnosis of glaucoma

A diagnosis of glaucoma was made based on both the structural changes (e.g., glaucomatous optic disc cupping or RNFL defects) and the presence of glaucomatous VF loss on standard automated perimetry (SAP)^35^, which was defined as the consistent presence of a cluster of 3 or more nonedge points on a pattern deviation plot with P < 5%, including one or more with P < 1%, a pattern standard deviation (PSD) < 5% or glaucoma hemifield test results outside the normal limits^36^, and on the presence of glaucomatous optic disc cupping (e.g., neuroretinal rim thinning, notching, excavation) or RNFL defects. VF defects had to be repeatable on at least 2 consecutive reliable tests (false positive/negatives < 15%, fixation losses < 15%)^37^. The appearance of the optic disc on optic disc photography and the RNFL on red-free RNFLP were evaluated by two glaucoma specialists (JL, HJC) who were blinded to all other information on the eyes. If the opinions on the diagnosis of glaucoma differed, the final judgment was made by consensus.

The control subjects had an IOP ≤ 21 mmHg with no history of increased IOP, absence of glaucomatous disc appearances or RNFL defects, and a normal VF on SAP.

### Statistical analysis

Unpaired *t*-tests and chi-square tests were performed to compare baseline clinical characteristics between healthy and glaucomatous eyes. A paired t-test was used for comparison of IOP and CCT measurements acquired from different types of equipment. Intraclass correlation coefficients (ICCs) were calculated to evaluate the intrasession repeatability of the T-CCT and T-IOP measurements and the intersession reproducibility of the T-CCT measurement. Considering the long-term IOP fluctuation, intersession reproducibility was analyzed only for T-CCT. Intersession reproducibility was calculated for the subjects who underwent T-CCT measurements three times within an interval of 6 months. Pearson correlation analysis and Bland–Altman analysis were used to assess the correlations and agreement. For the Bland–Altman plots, the bias with 95% confidence interval (CI) was calculated for T-IOP relative to G-IOP. The intersession reproducibility of CCT from three visits was additionally evaluated by CoV as a normalized SD, as shown in

$$CoV=\frac{SD}{mean} \times 100 \left(\%\right).$$

The smaller CoV means better reliability and the instruments with a CoV < 10% are generally regarded as having high repeatability, and a CoV < 5% indicates very high repeatability^38^. All statistical analyses were performed using R version 3.4.0. The data are presented as the mean ± standard deviation, and the level of statistical significance was P < 0.05.

## Supplementary Information

Supplementary Information.

## Data Availability

The dataset generated during the current study is available from the corresponding author on reasonable request.
