# Supplementary material for: Accuracy and reliability of measurements obtained with a noncontact tono-pachymeter for clinical use in mass screening
Source: Sci Rep. 2021 Apr 26;11:8900. doi: 10.1038/s41598-021-88364-8 (PMC8076298; doi:10.1038/s41598-021-88364-8)
Supplement: Supplementary file 1 — Supplementary Information. [file 41598_2021_88364_MOESM1_ESM.pdf]

Accuracy and Reliability of Measurements Obtained with a Noncontact Tono-Pachymeter for Clinical Use in Mass Screening

Jinho Lee, Hyuk Jin Choi

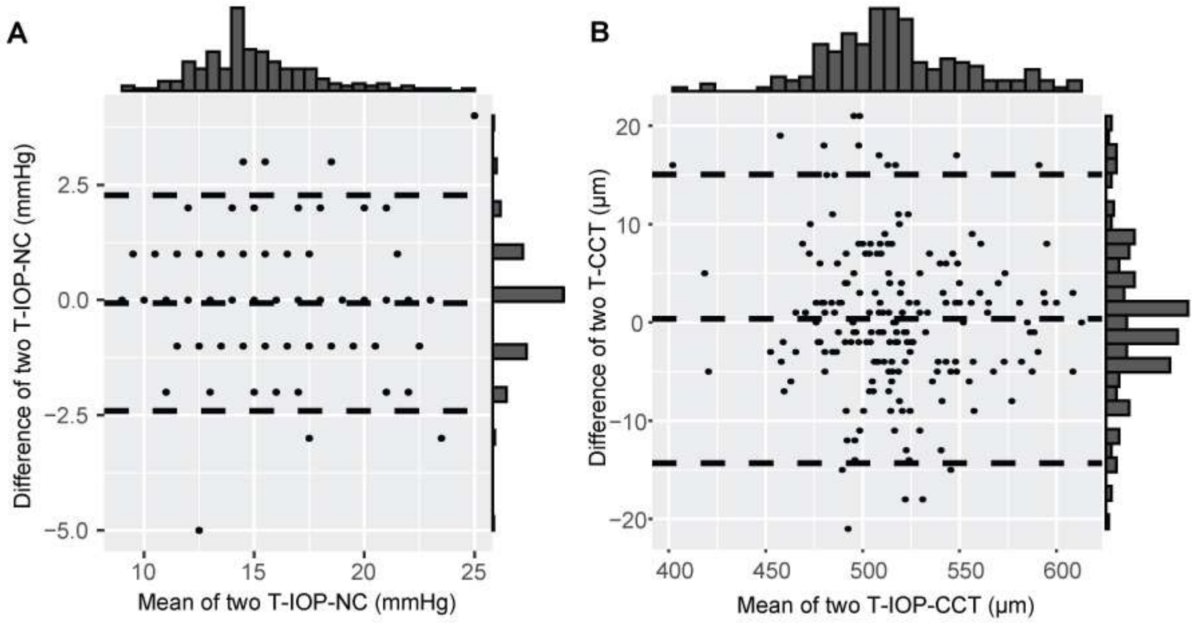

Supplemental Figure S1. Bland-Altman plots showing the intrasession repeatability of Topcon CT-1P. The dashed line in the center indicates the mean difference between both measurements. The upper and lower dashed lines are the 95% limits of agreement (LoA).

(A) Intrasession repeatability of unadjusted IOP (T-IOP-NC). There was no systemic bias between two IOP measurements (mean bias 0.68 mmHg, 95% CI [-0.24, 0.10]). 95% limit of agreement was [-2.40, 2.27 mmHg].

(B) Intrasession repeatability of CCT measured with CT-1P (T-CCT). There was no systemic bias between two CCT measurements (mean bias 0.37 µm, 95% CI [-0.69, 1.42]). 95% limit of agreement was [-14.3, 15.0 mmHg].

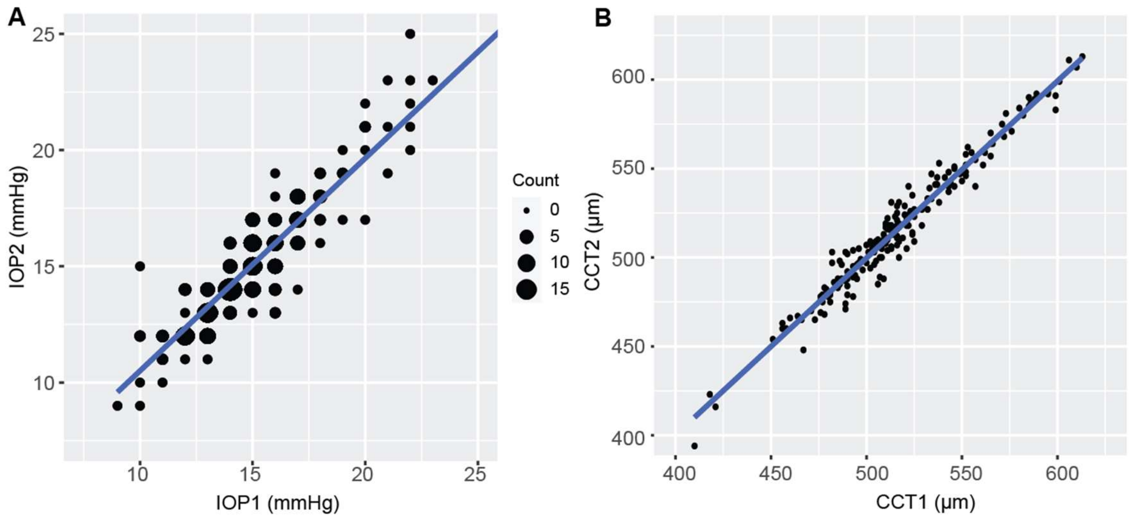

Supplemental Figure S2. Scatterplot showing the correlation between two measurements at same visit taken with a Topcon CT-1P noncontact tonopachymeter. (A) CCT-unadjusted IOP showed excellent relative reliability for intrasession measurements (ICC 0.91, 95% CI [0.89, 0.92]; P < 0.001). (B) T-CCT also showed excellent intrasession repeatability (ICC 0.98, 95% CI [0.98, 0.98]; P < 0.001).
